# Acquisition of resistance to trastuzumab in gastric cancer cells is associated with activation of IL-6/STAT3/Jagged-1/Notch positive feedback loop

**DOI:** 10.18632/oncotarget.3241

**Published:** 2014-12-31

**Authors:** Zhengyan Yang, Liang Guo, Dan Liu, Limin Sun, Hongyu Chen, Que Deng, Yanjun Liu, Ming Yu, Yuanfang Ma, Ning Guo, Ming Shi

**Affiliations:** ^1^ Institute of Basic Medical Sciences, Beijing, P.R. China; ^2^ Laboratory of Cellular and Molecular Immunology, Medical School of Henan University, Kaifeng, P.R. China

**Keywords:** trastuzumab, gastric cancer, STAT3, Notch

## Abstract

In the present study, we demonstrate that prolonged treatment by trastuzumab induced resistance of NCI-N87 gastric cancer cells to trastuzumab. The resistant cells possessed typical characteristics of epithelial to mesenchymal transition (EMT)/cancer stem cells and acquired more invasive and metastatic potentials both *in vitro* and *in vivo*. Long term treatment with trastuzumab dramatically inhibited the phosphorylation of Akt, but triggered the activation of STAT3. The level of IL-6 was remarkably increased, implicating that the release of IL-6 that drives the STAT3 activation initiates the survival signaling transition. Furthermore, the Notch activities were significantly enhanced in the resistant cells, companied by upregulation of the Notch ligand Jagged-1 and the Notch responsive genes Hey1 and Hey2. Inhibiting the endogenous Notch pathway reduced the IL-6 expression and restored the sensitivities of the resistant cells to trastuzumab. Blocking of the STAT3 signaling abrogated IL-6-induced Jagged-1 expression, effectively inhibited the growth of the trastuzumab resistant cells, and enhanced the anti-tumor activities of trastuzumab in the resistant cells. These findings implicate that the IL-6/STAT3/Jagged-1/Notch axis may be a useful target and that combination of the Notch or STAT3 inhibitors with trastuzumab may prevent or delay clinical resistance and improve the efficacy of trastuzumab in gastric cancer.

## INTRODUCTION

Trastuzumab, a therapeutic monoclonal antibody directed to the human epidermal growth factor receptor-2 (Her2), has been used as standard therapy in advanced Her2-positive breast cancers. It is also indicated in Her2-positive advanced gastric cancers in combination with chemotherapy. The international phase III trial of trastuzumab for gastric cancer showed a clinically and statistically significant benefit in terms of response rate, median progression-free survival, and median overall survival. Currently, trastuzumab is approved for the clinical treatment of gastric cancer patients with Her2-positive tumors [1, 2].

However, resistance to trastuzumab is common in both breast and gastric cancers. Her2 is overexpressed in ~25% of gastric cancer patients, who would supposedly benefit from trastuzumab therapy [3]. Unfortunately, the response rate to trastuzumab among the patients is only 12.8%, which is much lower than that previously reported in breast cancer [4]. Currently, much of what we know about the resistant mechanisms associated with trastuzumab, including hyperactivation of the phosphatidylinositol-3-kinase (PI3K) pathway, deficiency of phosphatase and tensin homolog, an inhibitor of the PI3K/AKT pathway, and mutation in the catalytic subunit α of PI3K [5-12], comes from the researches in breast cancer. However, the molecular mechanisms of intrinsic or acquired trastuzumab resistance in gastric cancer have not been extensively explored.

Tumor cells frequently display initial sensitivity to anti-tumor therapeutic drugs, but acquire resistance during the treatment. Several recent studies demonstrated that long-term treatment of tumor cells with chemotherapeutic drugs (such as 3-aminobenzamide, doxorubicin, and cisplatin) *in vitro* resulted in enrichment of the cell population with the characteristics of cancer stem cell (CSC) (the expression of stemness-related genes, high clonogenicity, self-renewal capacity, resistance to the cytotoxic effect of chemotherapy, and, critically, the ability to initiate the original tumors upon transplantation in immunodeficient mice). CSCs could also be enriched in xenogeneic tumors following chemotherapy or arise from differentiated cancer cells through epithelial to mesenchymal transition (EMT). The importance of EMT in tumor invasion, metastasis, and drug resistance has been increasingly recognized [13-15]. So far, it has been unknown that whether and how the phenotypes and biobehaviors of gastric cancer cells resistant to trastuzumab are altered.

Here, we provide evidence showing that acquisition of trastuzumab resistance is associated with the formation of EMT/CSC phenotype and transition of survival signaling through activating IL-6/STAT3/Jagged-1/Notch positive feedback signaling loop in gastric cancer cells.

## RESULTS

### Trastuzumab resistance is associated with EMT in gastric cancer cells

**Figure 1:**
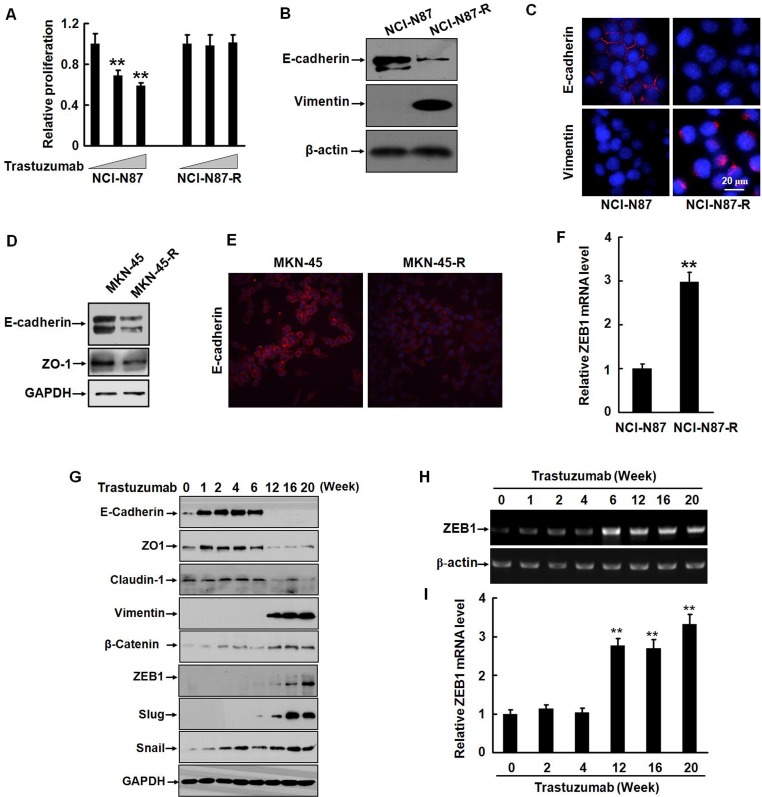
Trastuzumab resistance is associated with EMT in gastric cancer cells A, NCI-N87 and NCI-N87-R cells were cultured in 96-well plates with an initial cell density of 4 × 10^3^/well in DMEM containing 0, 5, or 10 μg/ml trastuzumab for five days. The *in vitro* proliferation activities were measured by CCK8 assays. B, The expression of E-cadherin and vimentin in NCI-N87 and NCI-N87-R cells was analyzed by Western blot. C, NCI-N87 and NCI-N87-R cells were labeled with the rabbit monoclonal antibodies against E-cadherin and vimentin. Binding was detected by Alexa fluor 549-labeled secondary antibody. Nuclei were stained with 1 μg/ml DAPI. The cells were observed under a laser scanning confocal microscope. Bar = 20 μm. D, The expression of E-cadherin and ZO-1 in MKN-45 and MKN-45-R cells was analyzed by Western blot. E, The expression of E-cadherin in MKN-45 and MKN-45-R cells was analyzed by immunofluorescence. F, The expression of the ZEB1 mRNA was detected by real-time RT-PCR. G, NCI-N87 cells were cultured in increasing concentration of trastuzumab and the expression of the epithelial and mesenchymal markers was analyzed by Western blot at the indicated time points. H and I, The expression of ZEB1 mRNA was detected by RT-PCR (H) and real-time RT-PCR (I) at the indicated time points after trastuzumab treatment. These experiments were repeated in duplicate. ** *P*<0.01.

To model the development of acquired trastuzumab resistance in patients, we treated Her2-overexpressing human gastric cancer cells (NCI-N87 and MKN-45) with increasing doses of trastuzumab for eight months and obtained the trastuzumab-resistant sublines NCI-N87-R and MKN-45-R. Compared with parental NCI-N87 cells, NCI-N87-R cells exhibited remarkable resistance to trastuzumab *in vitro* (Fig. 1A). Loss of an epithelial marker E-cadherin expression is a hallmark of EMT. We observed that the level of E-cadherin was dramatically downregulated and a mesenchymal marker vimentin, which was negative in the parental cells, upregulated in the resistant cells (Fig. 1B and 1C). Similar data were also observed in MKN-45 cells (Fig. 1D and 1E). In addition, an important EMT regulator, E-cadherin transcriptional repressor ZEB1 was also upregulated (Fig. 1F), suggesting that trastuzumab resistant cells underwent a phenotypic conversion.

To explore the mechanisms of trastuzumab resistance in gastric cancer cells, we reperformed the experiment. Fig. 1G shows that following exposure of NCI-N87 cells to 2.5 μg/ml trastuzumab for one week, the expression of the epithelial markers, including E-cadherin and zona occludens-1 (ZO-1, a critical regulator of epithelial tight junctions) was significantly increased. Another epithelial marker, claudin-1 (an adhesion molecule) was not changed. Increased levels of E-cadherin and ZO-1 were sustained for six weeks. Interestingly, the expression of these molecules was either disappeared or declined subsequently. Concomitant with the decrease of the epithelial markers, the expression of a mesenchymal marker vimentin and EMT regulatory factors ZEB1 and Slug were markedly increased by six weeks of treatment with trastuzumab. The expression of these mesenchymal markers was maintained at high levels up to 12 weeks. The upregulation of ZEB1 at the transcription level was also detected (Fig. 1H and 1I). Noticeably, upregulation of Snail, a transcription repressor of E-cadherin was coincident with the elevation of the E-cadherin and ZO-1 levels, implicating a negative feedback mechanism of regulating the expression of the epithelial markers by Snail. β-catenin is an essential component of adherent junctions. The E-cadherin-catenin complex modulates cell-cell adhesion and cell migration. It has been reported that activation of the β-catenin pathway is critical for the maintenance of EMT and that β-catenin was involved in regulation of EMT-like behaviors in tamoxifen-resistant MCF-7 human breast cancer cells [16, 17]. We noticed that the β-catenin level was prominently elevated after trastuzumab treatment for six weeks (Fig. 1G). These data suggest that gastric cancer cells underwent EMT during development of trastuzumab resistance.

### Trastuzumab resistant cells have high potentials of migration, invasion, tumorigenesis, and metastasis

**Figure 2:**
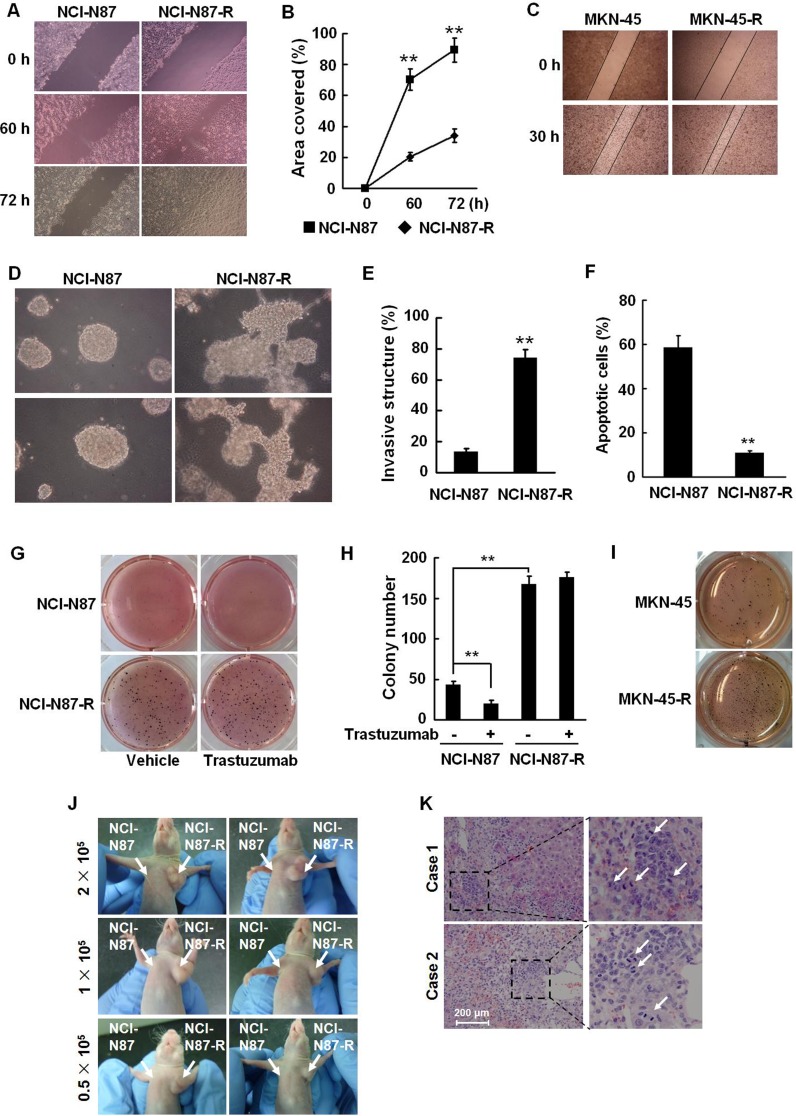
Trastuzumab resistant cells are highly aggressive A and B, NCI-N87 and NCI-N87-R cells were cultured in six-well plates. The confluent cell monolayers were wounded by scraping and the closure of the wounded areas was monitored. The images of the wounds were taken at the indicated time points (A) and the rate of cell migration by measuring the distance traveled toward the center of the wound was calculated (B). C, Migration activities of MKN-45 and MKN-45-R cells were analyzed by scratch wound assays and the images of the wounds were taken at 0 and 30 h. D and E, NCI-N87 and NCI-N87-R cells were suspended in a mixture of Matrigel matrix and culture medium (1:24, v/v) and then layered onto solidified Matrigel. The images were taken after 10 days of culture (D). The invasive structures in 3D cell cultures were counted in five random areas under a light microscope (E). F, NCI-N87 and NCI-N87-R cells were cultured in the plates coated with PolyHEMA for 72 h and cellular apoptosis was analyzed by flow cytometry using Annexin V-FITC detection kit. G and H, 5 × 10^3^ NCI-N87 and NCI-N87-R cells were suspended in DMEM containing 10% FBS and 0.35% agar and plated on top of the bottom layer of 0.6% agarose in six-well plates. 5 μg/ml trastuzumab or PBS was added twice a week. Colonies were stained with MTT, photographed (G) and counted (H) after 16 days. I, Soft agar colony formation assays were performed in MKN-45 and MKN-45-R cells and photographs taken. These experiments were performed in duplicate. J, 2 × 10^5^, 1 × 10^5^, and 0.5 × 10^5^ NCI-N87 and NCI-N87-R cells were injected subcutaneously in the left and right upper flank of the mice, respectively. 20 days following tumor cell implantation, the mice bearing tumors were photographed. K, 2 × 10^6^ NCI-N87 or NCI-N87-R cells were injected subcutaneously in the right upper flank of the mice. 40 days following tumor implantation, the livers of the mice were autopsied and paraffin embedded. The sections were stained with HE. White arrows point to mitotic cells. Bar = 200 μm ** *P*<0.01.

EMT process is generally accompanied with an enhancement in cellular invasion and migration [18, 19]. We evaluated the migration activities of the resistant cells by wound healing assays. Compared to their parental cells, NCI-N87-R and MKN-45-R cells had significantly higher migration activities, as evidenced by the fact that a more rapid and complete wound closure was observed in these cells (Fig. 2A, 2B, and 2C). We utilized a more physiological approach, three-dimensional (3D) cell culture to assess tumor cell invasion. As shown in Fig. 2D and 2E, NCI-N87-R cells displayed a remarkable capacity to invade through a reconstituted basement membrane (Matrigel).

Anchorage to the extracellular matrix (ECM) promotes cell survival whereas loss of cell adhesion triggers apoptosis, a process termed anoikis, in epithelial cells. During EMT cellular sensitivity to anoikis is compromised. We cultured the cells in the plates coated with PolyHEMA in the media containing 1% serum. The cells grew predominantly as a single-cell suspension. NCI-N87 cells underwent anoikis after loss of detachment for 72 h, with the anoikis rates of approximately 60%. Conversely, NCI-N87-R cells exhibited a significant resistance to anoikis and the anoikis rate was only about 10% (Fig. 2F), indicating that the growth of the cells is in an anchorage-independent manner. In addition, NCI-N87-R and MKN-45-R cells could more actively form colonies in soft agar, compared with their parental cells (Fig. 2G, 2H, and 2I). In the presence of trastuzumab, the proliferation of NCI-N87-R cells was not affected. In contrast, when trastuzumab was added, NCI-N87 cells almost stopped growing and colonies formed by NCI-N87 cells were much smaller and less (Fig. 2G and 2H). These data suggest that trastuzumab resistant cells acquired malignant traits.

We then evaluated the tumorigenic potential of the cells in an animal model. Several independent experiments were performed with NCI-N87 and NCI-N87-R cells. 2 × 10^5^, 1 × 10^5^, and 0.5 × 10^5^ NCI-N87 and NCI-N87-R cells were subcutaneously injected into athymic nude mice. When minimal numbers of cells (0.5 × 10^5^) per mouse were injected, NCI-N87-R cells formed tumors within 10 days. The implantation of greater numbers of NCI-N87-R cells produced larger tumors. However, even maximal numbers of NCI-N87 cells (2 ×10^5^) were injected, no tumors were generated within 20 days (Fig. 2J). To further confirm it, the mice were divided into six groups randomly and each group contained five mice. 2 × 10^5^, 0.5 × 10^5^, or 5 × 10^3^ NCI-N87 and NCI-N87-R cells were injected subcutaneously to the mice. The tumors were detected in the mice injected with 2 × 10^5^, 0.5 × 10^5^, or 5 × 10^3^ NCI-N87-R cells within 7, 10 or 20 days, respectively. No tumor was observed within 20 days in the mice injected with an equal number of NCI-N87 cells. Tumor formation only occurred after implantation of 2 × 10^5^ NCI-N87 cells for 36 days (Supplementary Fig. S1A and S1B). The data suggest that the resistant cells may possess the characteristics of tumor-propagating cells.

We examined liver metastases from the xenografts in nude mice. As displayed in Fig. 2K, subcutaneous implantation of 5×10^6^ NCI-N87-R cells resulted in spontaneous liver metastases in nude mice and vigorous mitosis was observed in the metastatic loci. NCI-N87 xenografts did not develop liver metastasis. We detected the expression of vimentin in the xenograft tumors by immunohistochemistry. The data demonstrate that vimentin is predominantly expressed in mesenchymal cells, which were around NCI-N87 xenograft tumor tissues. However, the expression of vimentin was found to localize in both tumor and mesenchymal cells in the NCI-N87-R xenograft tumors (Supplementary Fig. S1C). In addition, the expression of E-cadherin was lost and ZEB-1 significantly upregulated in NCI-N87-R xenograft tumors (Supplementary Fig. S1D). These data confirm that trastuzumab resistant cells have higher potentials of migration, invasion, tumorigenesis, and metastasis.

### Trastuzumab resistant gastric cancer cells acquire the phenotype of cancer stem-like cells

**Figure 3:**
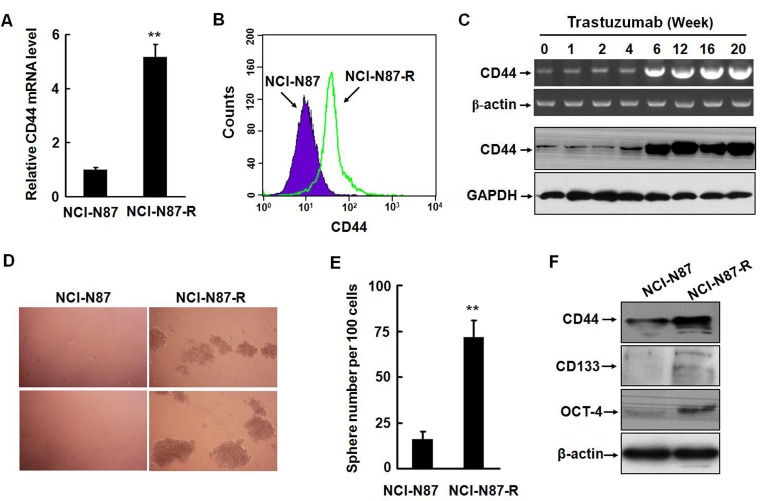
Trastuzumab resistant gastric cancer cells acquire the phenotype of cancer stem-like cells A and B, The expression of CD44 in NCI-N87 and NCI-N87-R cells was detected by real-time RT-PCR (A) and flow cytometry (B). C, The alteration of CD44 at the mRNA and protein levels was analyzed by RT-PCR and Western blot at the indicated time points after trastuzumab treatment. D and E, The *in vitro* self-renewal capacities of NCI-N87 and NCI-N87-R cells were assessed by spheroid colony formation assays by culturing the cells under nonadherent conditions with serum-free media. After two weeks of culture, spheres were photographed (D) and sphere number per 100 cells was counted (E). F, The expression of CD44, CD133, and OCT-4 was analyzed by Western blot in NCI-N87 and NCI-N87-R cells. The experiments were performed at least twice. ** *P*<0.01.

It is known that EMT can endow cells with stem-cell like characteristics, such as self-renewal, differentiation, and resistance to chemotherapy or radiotherapy. Several previous studies demonstrated that CD44-positive gastric cancer cells possessed the features of CSC. In severe combined immunodeficiency mice, CD44-positive gastric cancer cells are highly tumorigenic and resistant to chemotherapeutics or radiation [20, 21]. CD44 is a cell surface transmembrane receptor for hyaluronic acid, one of the extracellular matrix components and participates in regulating cell-cell interaction, cell adhesion, and migration. CD44 is also a known downstream target of Wnt/β-catenin pathway and expressed in a variety of solid tumors including gastric cancer [22]. We analyzed the expression of CD44 in NCI-N87 and NCI-N87-R cells by real-time RT-PCR and flow cytometry. The level of CD44 in parental NCI-N87 cells was very low, which is consistent with the data presented in a previous study [18]. However, NCI-N87-R cells expressed significantly higher level of CD44 at both mRNA and protein levels (Fig. 3A and 3B). Noticeably, the expression of the CD44 mRNA and protein was upregulated at the sixth week of postinduction with trastuzumab (Fig. 3C and Supplementary Fig. S2A) and elevation of the CD44 level was accompanied by upregulation of key EMT inducers, Slug and ZEB1 (Fig. 1G).

To determine the *in vitro* self-renewal capacity of NCI-N87-R cells, we performed spheroid colony formation assays by culturing NCI-N87-R cells under nonadherent conditions with serum-free media. The growth of spherical colonies, which is considered as an indication of self-renewal ability, was observed after culturing for two weeks. As expected, NCI-N87-R cells generated significantly larger and more spheroid colonies than NCI-N87 cells (Fig. 3D and 3E). Based on previous published reports regarding CSC markers in gastric cancer cells, we also examined other stemness markers, which are highly expressed in gastric cancer, including CD133 and the octamer-binding transcription factor 4 (OCT4) that is involved in regulating pluripotency and self-renewal maintenance of embryonic stem cells. Fig. 3F shows that NCI-N87-R cells expressed higher levels of CD133 and OCT4 than parental cells. Enhanced expression of OCT4 was also observed in MKN-45-R cells (Supplementary Fig. S2B). Intriguingly, NCI-N87-R cells maintained this phenotype even in the absence of trastuzumab in subsequent passages. As mentioned above, the NCI-N87-R cells yielded tumors with as few as 5 × 10^3^ cells in all mice. These data clearly indicate that trastuzumab resistant NCI-N87-R cells acquire the phenotype of cancer stem-like cells.

### Survival signaling was shifted in trastuzumab resistant NCI-N87-R cells

**Figure 4:**
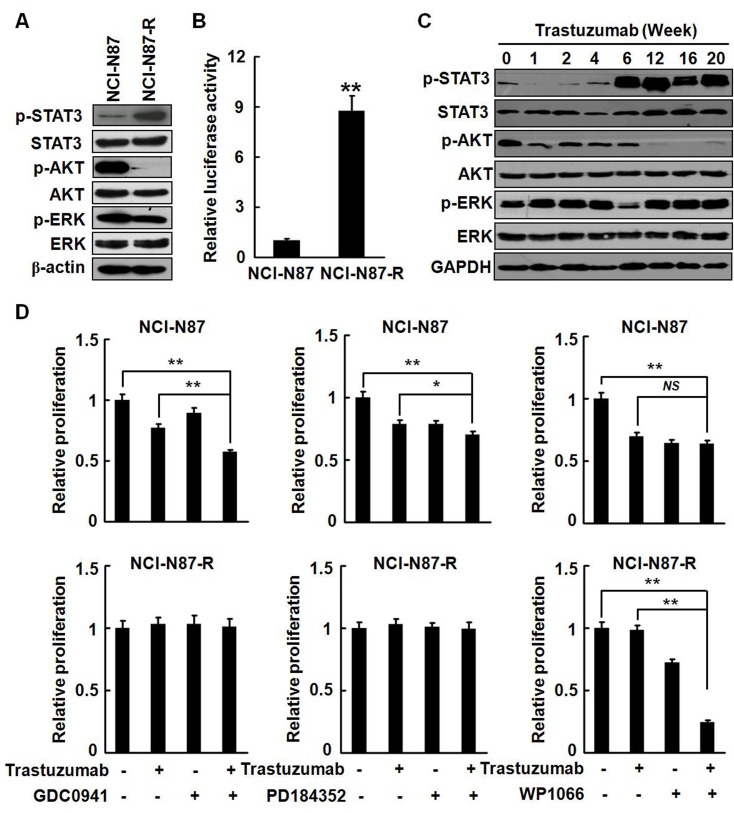
Survival signaling was shifted in NCI-N87-R cells A, The phosphorylation of STAT3, Akt, and ERK in NCI-N87 and NCI-N87-R cells was analyzed. B, The STAT3 transcription activities were analyzed by luciferase assays. C, NCI-N87 cells were cultured in increasing concentration of trastuzumab. The Phosphorylation of STAT3, Akt, and ERK was assessed at the indicated time points. D, NCI-N87 and NCI-N87-R cells were cultured in DMEM containing 10 μg/ml trastuzumab with or without 100 nM GDC0941, 1 μM PD184352 or 2.5 μM WP1066, respectively. The *in vitro* proliferation activities were analyzed by CCK8 assays at the indicated time points. The experiments were performed in duplicate. ** *P*<0.01; * *P*<0.05.

Activation of the PI3K pathway, on which Her2 signaling is highly dependent, has been implicated as a key mediator of trastuzumab resistance in breast cancer [23]. To explore the signaling mechanisms of trastuzumab resistance in gastric cancer, we examined the phosphorylation status of Akt, ERK, and STAT3, which are well known to be major cell survival pathways mediated by Her2. In parental NCI-N87 cells, the phosphorylation levels of Akt and ERK were high, whereas the phoshorylation of STAT3 was barely detected. The ERK phosphorylation status was not changed in NCI-N87-R cells. Surprisingly, phosphorylated STAT3 level was dramatically increased in NCI-N87-R cells, but the phosphorylation of Akt was almost completely disappeared (Fig. 4A), implicating that the survival signaling on which the growth of parental NCI-N87 cells depended may be shifted. To evaluate the STAT3 transcription activities, NCI-N87 and NCI-N87-R cells were co-transfected with the STAT3 reporter plasmid and the transcriptional activities of STAT3 were determined by luciferase assays. Fig. 4B demonstrates that the luciferase activities in NCI-N87-R cells were significantly increased by nearly nine-folds compared to NCI-N87 cells. Time course analyses show that the phosporylation of Akt was remarkably decreased after trastuzumab treatment for one week and then hardly detectable after 12 weeks, reflecting the inhibitory effect of trastuzumab on the Akt activation. Noticeable, the STAT3 phosphorylation was initiated at the sixth week of posttreatment with trastuzumab and remained persistently high up to 20 weeks (Fig. 4C). Elevated phosphorylation of STAT3 was also seen in MKN-45-R cells (Supplementary Fig. S2B).

To determine the importance of the STAT3 signaling pathway in the growth of the resistant cells, we treated the cells with GDC0941 (a potent inhibitor of PI3Kα/δ), PD184352 (an ATP non-competitive MEK1/2 inhibitor), and WP1066 (an inhibitor of Jak2/STAT3). Fig. 4D shows that 10 ng/ml of trastuzumab could effectively suppress the growth of NCI-N87 cells. All inhibitors appeared to have inhibitory effects on the growth of the parental cells. GDC0941 could enhance the antiproliferation activities of trastuzumab in the parental cells. However, GDC0941 and PD184352 did not affect the proliferation of NCI-N87-R cells, but treatment with WP1066 significantly inhibited the growth of NCI-N87-R cells. Combined treatment with trastuzumab and WP1066 resulted in a more dramatic growth inhibition of the resistant cells. Taken together, the data indicate that the survival signaling is shifted in trastuzumab resistant NCI-N87-R cells and that STAT3 plays a predominant role in the survival and proliferation of NCI-N87-R cells.

### IL-6 autocrine is involved in development of trastuzumab resistance in NCI-N87 cells

**Figure 5:**
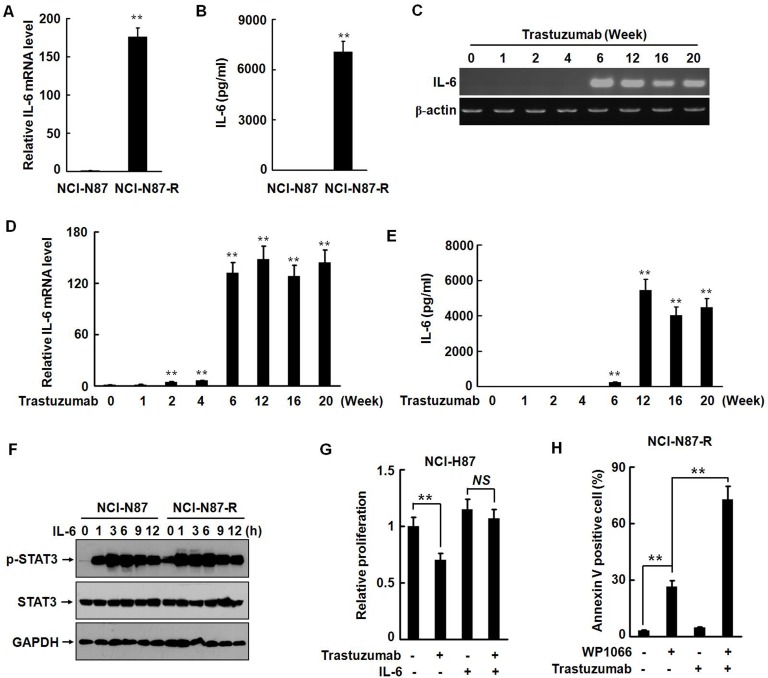
IL-6 autocrine is involved in development of trastuzumab resistance in NCI-N87 cells A and B, The expression and secretion of IL-6 in NCI-N87 and NCI-N87-R cells were analyzed by real-time RT-PCR (A) and ELISA (B). C to E, The expression and secretion of IL-6 were analyzed by RT-PCR (C), real-time RT-PCR (D), and ELISA (E) at the indicated time points after trastuzumab treatment. F, NCI-N87 and NCI-N87-R cells were treated with 10 ng/ml of IL-6. The phosphorylation of STAT3 was analyzed at the indicated time points. G, NCI-N87 cells were cultured in DMEM containing 10 μg/ml trastuzumab with or without 10 ng/ml of IL-6. The *in vitro* proliferation activities were analyzed by CCK8 assays. H, NCI-N87-R cells were treated with 2.5 μM WP1066 and 10 μg/ml trastuzumab. Cellular apoptosis was assessed by flow cytometry using Annexin V-FITC detection kit. The experiments were performed at least twice. ** *P*<0.01.

STAT3 is a key player in mediating inflammation-driven tumorigenesis. Inflammatory cytokines, such as IL-1β, TNF-α, and IL-6, activate STAT3 either directly or indirectly [24, 25]. A previous study demonstrates that development of trastuzumab resistant in breast cancer cells is mediated by activation of an IL-6 inflammatory feedback loop [10]. Gastric mucosal inflammation has been associated with tumorigenesis of gastric cancer. IL-6 as an important activator of oncogenic STAT3 links inflammation to malignant transformation by activating several inflammation-related signaling pathways and inducing EMT. Therefore, we examined the effects of trastuzumab treatment on the expression and secretion of IL-6 in parental and trastuzumab resistant NCI-N87-R cells. In parental NCI-N87 cells there was no detectable expression and secretion of IL-6. However, the expression of the IL-6 mRNA was dramatically increased by more than 150-folds (Fig. 5A) and the secretion level of IL-6 as high as 7000 pg/ml in NCI-N87-R cells (Fig. 5B). We noticed that the temporal spatial expression pattern of IL-6 was coincident with the phenotypic transition of NCI-N87 cells and activation of STAT3 (Fig. 5C to 5E). In the parental cells, the level of phosphorylated STAT3 was very low, but STAT3 was constitutively activated in NCI-N87-R cells. Addition of recombinant IL-6 caused a marked increase of phosphorylated STAT3 in both NCI-N87 and NCI-N87-R cells (Fig. 5F). The anti-proliferation effect of trastuzumab was significantly abrogated by IL-6 in parental NCI-N87 cells (Fig. 5G). Treatment with WP1066 alone dramatically caused apoptosis of NCI-N87R cells and combination of trastuzumab with WP1066 induced a much stronger pro-apoptosis effect (Fig. 5H). Together, these results suggest that IL-6 autocrine is involved in development of trastuzumab resistance in NCI-N87 cells.

### An IL-6/Notch positive feedback signaling loop exists in NCI-N87-R cells

**Figure 6:**
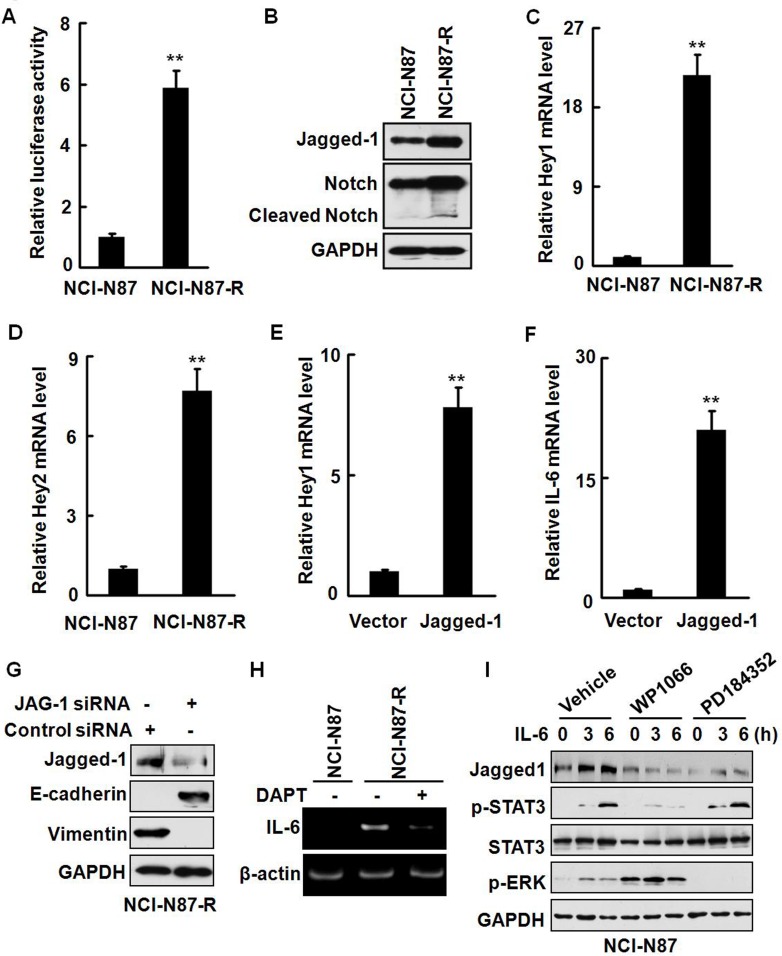
An IL-6/Notch positive feedback signaling loop exists in NCI-N87-R cells A, The Notch reporter activities in NCI-N87 and NCI-N87-R cells were analyzed by luciferase assays. B, The expression of Jagged-1 and cleavage of the Notch reporter were analyzed. C and D, The expression of the Hey1 (C) and Hey2 (D) mRNA was detected by real-time RT-PCR. E and F, NCI-N87 cells were transfected with the plasmid expressing Jagged-1. The expression of Hey1 (E) and IL-6 (F) at the mRNA levels was analyzed by real-time RT-PCR. G, NCI-N87-R cells were transfected with the *JAG-1* siRNA or control siRNA and the expression of Jagged-1, E-cadherin, and vimentin was analyzed by Western blot. H, NCI-N87 and NCI-N87-R cells were treated with 2 μM DAPT. The expression of the IL-6 mRNA was analyzed by RT-PCR. I, NCI-N87 cells were treated with 10 ng/ml IL-6 in the presence or absence of 2.5 μM WP1066 or 1 μM PD184352. The expression of Jagged-1 and phosphorylation of STAT3 and ERK were detected at the indicated time points. The experiments were performed at least twice. ** *P*<0.01.

The oncogenic functions of the Notch signaling have been well documented and the roles of the Notch signaling in various stem and early progenitor cells have also been recognized [26]. Several recent studies indicate that IL-6 is a novel Notch target in breast cancer cells and enhanced Notch signaling upregulates the IL-6 expression, leading to activation of autocrine and paracrine Jak/STAT signaling. On the other hand, the IL-6 signaling can drive the expression of Notch-dependent genes. It has also been reported that IL-6 induces malignant phenotypes in Notch-expressing stem/progenitor cells from human breast cancer [27-30]. To determine whether the Notch signaling is activated in the resistant cells, we transfected NCI-N87 and NCI-N87-R cells with a Notch reporter plasmid and measured the luciferase activities. Fig. 6A shows that the Notch reporter activities were significantly higher (approximately six folds) in NCI-N87-R cells than in NCI-N87 cells. The expression of the Notch ligand Jagged-1 was also remarkably enhanced in NCI-N87-R and MKN-45-R cells (Fig. 6B and Supplementary Fig. S2B). Moreover, Notch cleavage products that are indicative of the Notch activation were observed in NCI-N87-R cells (Fig. 6B). In addition, the expression of the Notch target genes Hey1 and Hey2 at the mRNA levels was remarkably increased by about 20 and seven folds, respectively (Fig. 6C and 6D). We noticed that elevation of the Hey1 mRNA expression (Supplementary Fig. S3A) was coincident with the upregulation of the IL-6 mRNA expression (Fig. 5D), whereas the level of Hey2 was progressively elevated (Supplementary Fig. S3B). Ecotopic overexpression of Jagged-1 [31] resulted in a dramatic upregulation of the Hey1, Hey2, and IL-6 at the mRNA level (Fig. 6E, 6F, and Supplementary Fig. S4A). Knock-down of the Jagged-1 expression in NCI-N87-R cells remarkably restored the expression of E-cadherin and suppressed the expression of vimentin (Fig. 6G). These data indicate that the activation of the Jagged-1/Notch signaling pathway is involved in the development of trastuzumab resistance in gastric cancer cells.

Notch signaling is initiated by γ-secretase-dependent cleavage of the Notch receptor following binding of the Notch receptors to their cognate ligands [32]. We observed that treatment of NCI-N87-R cells with γ-secretase inhibitor DAPT effectively repressed the expression of IL-6 (Fig. 6H), demonstrating that activation of the Notch signaling is associated with the production of IL-6 in NCI-N87-R cells. Il-6 stimulation upregulated the expression of Jagged-1 in a time-dependent manner (Fig. 6I and Supplementary Fig. S4B). WP1066 but not PD184352 markedly inhibited IL-6-induced Jagged-1 expression (Fig. 6I). Although inhibition of the Notch pathway by γ-secretase inhibitor LY411575 alone did not markedly repress the proliferation of NCI-N87-R cells in 3D culture system, invasive potential of NCI-N87-R cells appeared to be reduced (Supplementary Fig. S5A). Simultaneous blocking of the STAT3 and Notch pathways by WP1066 and LY411575 almost completely inhibited the proliferation of NCI-N87-R cells in 3D culture system and also greatly suppressed self-renewal capacities (sphere formation) of NCI-N87-R cells (Supplementary Fig. S5A and S5B). Additionally, LY411575 also significantly restored the sensitivity of the resistant cells to trastuzumab (Supplementary Fig. S5C). These data demonstrate that IL-6/STAT3 and Jagged-1/Notch pathways synergize in induction of trastuzumab resistance in gastric cancer cells.

## DISCUSSION

The development of resistance is a major obstacle to trastuzumab-based treatment in both Her2-overexpressing breast and gastric cancers. Multiple mechanisms driving trastuzumab resistance have been proposed in breast cancer [33], but it is unclear whether similar mechanisms exist in gastric cancer [34-36]. Exploration of the molecular mechanisms and identification of the phenotypic identity of resistant cells will facilitate the discovery of novel targets and development of personalized therapies in gastric cancer.

In the present study, we obtained trastuzumab-resistant sublines from human gastric cancer cell lines NCI-N87 and MKN-45 by repetitive, dose-escalating trastuzumab treatment *in vitro*. The cell populations exhibited typical characteristics of EMT (loss or downregulation of epithelial markers E-cadherin and ZO-1 and gain of mesenchymal markers vimentin, ZEB1, Slug, and Snail) and acquired more invasive and metastatic potentials both *in vitro* and *in vivo* (invasively growth in 3D culture and spontaneous liver metastasis in nude mice). EMT can trigger reversion to a CSC-like phenotype. CSCs, which have tumor-initiating capability and high metastatic potential, tend to be resistant to conventional therapeutic strategies such as radiotherapy or chemotherapy. The emergence of EMT and CSCs by treatment with chemotherapeutic agents has been reported [14]. We found that the long-term trastuzumab treatment induced CSC-like properties in gastric cancer cells, including elevated expression of CD44, CD133, and OCT4, self-renewal (forming mammospheres), higher clonogenicity, and increased tumorigeniticy. These data indicate that prolonged treatment by trastuzumab induces EMT and CSC-like phenotype in gastric cancer cells, resulting in resistance to trastuzumab.

Tumors with aberrantly activated oncogenes are frequently “addicted” to the oncogene-associated signaling pathways [37]. In NCI-N87 cells, ERK and Akt are constitutively active, implicating that the ERK and PI3K/Akt pathways are major signaling pathways for survival of NCI-N87 cells. Prolonged treatment by trastuzumab dramatically inhibited the phosphorylation of Akt, but triggered the activation of STAT3, indicating that inhibition of the survival signaling, which parental NCI-N87 cells were addicted to, are compensated by activating the STAT3 signaling pathway. Activation of feedback compensation circuit following inhibition of survival signaling has been associated with drug resistance. The coupling of the PI3K/Akt signaling blocking and STAT3 activation is an important event that promotes survival of the resistant gastric cancer cells in the presence of trastuzumab. Several survival signaling pathways, such as mitogen-activated protein kinase (MAPK), PI3K/Akt, mammalian target of rapamycin (mTOR), and Notch pathways, have been linked to the drug resistance of conventional chemotherapy [38-42]. For example, ERK, c-Jun N-terminal kinase (JNK), and p38 kinase are activated following exposure to chemotherapeutics in a variety of cancer cells, such as melanoma, breast, colon, and hepatocellular cancer cells [43, 44]. Activation of the PI3K/Akt/mTOR signaling pathway is known to mediate resistance to both chemotherapy and molecularly targeted therapy in various cancers [45, 46]. A recent study demonstrates that inhibition of EGFR by erlotinib, an EGFR inhibitor results in activation of the STAT3 signaling in lung cancer cells [47]. We found that the level of IL-6 was extremely high after trastuzumab treatment for six weeks when the STAT3 signaling became predominant survival signaling pathway in the resistant gastric cancer cells, implicating that the release of IL-6 that drives the STAT3 activation in response to trastuzumab initiates the survival signaling transition, which contributes to resistance to trastuzumab via a feedback mechanism.

The previous studies suggested that the Notch signaling is involved in chemotherapy-triggered CSC transition. Activation of the Notch signaling is also associated with induction of EMT. It is reported that the Notch signaling is important for the maintenance of self-renewal in human CD34+ cord blood cells and cancer-initiating cells as well [48, 49]. IL-6 is also capable of expanding CSCs and inducing EMT [50]. A recent report identified IL-6 as a target of Notch in breast cancer cells. It was shown that activated Notch1 induced the IL-6 transcription via interaction with the IL-6 promoter [51]. In NCI-N87 cells stimulation by IL-6 induced the expression of Jagged-1, whereas overexpression of Jagged-1, in turn, promoted the expression of IL-6 that triggered the activation of STAT3. Blocking the STAT3 activation by WP1066 effectively abrogated IL-6-induced Jagged-1 expression, strongly inhibited the growth of the trastuzumab resistant cells, and enhanced the anti-tumor activities of trastuzumab in the resistant cells. The data suggest that autocrine release of IL-6 and activation of Notch may comprise a positive feedback loop [28, 30]. We observed that the Notch activities were significantly enhanced in the resistant cells, companied by a substantial increase in the expression of Jagged-1 and the Notch responsive genes Hey1 and Hey2. Inhibiting the endogenous Notch pathway by γ-secretase inhibitor DAPT reduced the IL-6 expression. However, the upregulation of IL-6 in NCI-N87-R cells could not be completely blocked by DAPT. A previous study showed that preclinical efficacy of γ-secretase inhibitor, RO4929097 could be abrogated by overexpression of IL-6 [52]. It was also reported that Src activity was markedly increased in trastuzumab resistant gastric cancer cells [53] and that activated Src was capable of interacting with and activating Stat3 in fibroblasts [54], implying that other signaling pathways may also be involved in development of trastuzumab resistance. Simultaneous inhibition of the STAT3 and Notch pathways greatly inhibited the malignant behaviors of NCI-N87-R cells. Additionally, inhibition of STAT3 or Notch signaling pathway significantly restored the sensitivity of the resistant cells to trastuzumab. The findings suggest that sustained activation of the Jagged-1/Notch signaling in gastric cancer cells elicits an aberrant release of IL-6, leading to the transition of survival signaling, phenotype conversion, and resistance to trastuzumab. Our data support the hypothesis that cancer cells may undergo adaptive changes after anti-tumor therapies (either chemotherapy or targeted therapy) and also demonstrate that the IL-6/STAT3/Jagged-1/Notch signaling pathway exerts critical functions in trastuzumab resistant gastric cancer cells.

In summary, our data indicate that prolonged treatment by trastuzumab induced resistance in Her2-overexpressing gastric cancer cells. Blocking of a major survival pathway by trastuzumab is associated with production of IL-6, compensatory activation of STAT3, induction of EMT/CSC-like phenotype, and survival of resistant cells in the presence of trastuzumab, revealing an important cell-protective feedback mechanism. These findings implicate that the IL-6/STAT3/Jagged-1/Notch axis may be a useful target and combination of the STAT3 or Notch inhibitors with trastuzumab may prevent or delay clinical resistance and improve the efficacy of trastuzumab in gastric cancer.

## MATERIALS AND METHODS

### Cell culture and treatment

Human gastric cancer cell lines NCI-N87 and MKN-45 are obtained from the American Type Culture Collection. The cells were maintained in Dulbecco's modified Eagle's medium (DMEM) containing 10% fetal bovine serum (FBS), penicillin (100 U/ml), and streptomycin (100 μg/ml) at 37°C under 5 % CO_2_. Trastuzumab resistant NCI-N87 (NCI-N87-R) or MKN-45 (MKN-45-R) cells were established by culturing the cells in increasing concentration of trastuzumab over a one year period and final concentration of trastuzumab was 10 μg/ml at the end of the period.

### Western blot

The following antibodies were used for immunoblotting: the antibodies against E-cadherin (Cell Signaling), vimentin (Cell signaling), ZO1 (Cell signaling), Claudin-1 (Cell Signaling), β-catenin (Cell Signaling), ZEB1 (Cell Signaling), Slug (Cell Signaling), Snail (Cell Signaling), p-STAT3 (Cell Signaling), STAT3 (Cell Signaling), p-AKT (Cell signaling), AKT (Cell signaling), p-ERK (Santa Cruz), ERK (Santa Cruz), CD133 (Abcam), OCT4 (Cell signaling), Jagged-1 (Cell signaling), and glyceraldehyde-3-phosphate dehydrogenase (GAPDH, Sungene Biotech).

### Immunofluorescence, immunohistochemistry, and confocal microscopy

The parental and trastuzumab resistant gastric cancer cells were labeled with the rabbit monoclonal antibodies against E-cadherin and vimentin. Bound antibodies were detected by Alexafluor 549-labeled (red) secondary antibody (Invitrogen). Nuclei were stained with 1 μg/ml DAPI (4,6-diamidino-2-phenylindole; Sigma). The cells were observed under a laser-scanning confocal microscope (LSM 510 META, ZEISS). The experiment was repeated in duplicate.

The xenograft tumor tissues were fixed and embedded in paraffin. Immunohistochemistry assay was performed as described previously [34]. The expression of vimentin was detected by the rabbit monoclonal antibody (Cell Signaling Technology). Images were taken under a microscope (Olympus) using the Spot insight image capture system CCD camera.

### Conventional and quantitative RT-PCR

The total RNA was isolated from NCI-N87 cells, which were cultured with the media containing 10 μg/ml trastuzumab, using TRIzol reagent (Invitrogen) at the indicated time points. cDNA were synthesized by using reverse transcription kit (Promega) in accordance with the manufacturer's instructions. The specific primers (Supplementary Table S1) were used to detect the mRNA expression of ZEB1, IL-6, Hey1, Hey2, and CD44. Amplification of β-actin with the primers (Supplementary Table S1) was used as the control. The conventional and quantitative RT-PCR experiments were conducted as described in our previous study. The experiments were performed three times independently.

### Proliferation assay

NCI-N87 and NCI-N87-R cells were cultured in 96-well plates with an initial cell density of 4 × 10^3^/well in DMEM containing 0, 5, or 10 μg/ml trastuzumab. To determine the roles of the STAT3, AKT, and ERK signaling in the development of trastuzumab resistance, cells were treated with 5 μM WP1066 (Jak2/STAT3 inhibitor, Calbiochem), 100 nM GDC0941 (PI3K inhibitor, Calbiochem), 10 μM LY411575 (selective γ-secretase inhibitor) or 1 μM PD184352 (MEK1/2 inhibitor, Calbiochem), respectively. After incubation for different time periods, the *in vitro* proliferation activities were measured by CCK8 assays following the manufacturer's instruction. The experiments were performed in duplicate.

### Scratch wound assay

NCI-N87 and NCI-N87-R cells were cultured in six-well plates. The confluent cell monolayers were wounded by scraping once horizontally and vertically with a 200 μl pipette tip and further incubated in DMEM with 0.5% FBS. The images of the wounds at different time points were captured using the Olympus CKX41 microscope system.

### 3D cell culture

3D cell cultures were performed as described in the previous study [55]. Briefly, NCI-N87 and NCI-N87-R cells (1 × 10^5^/ml) were suspended in a mixture of Matrigel matrix (BD-Biosciences) and culture medium (1:24, v/v) and then layered onto solidified Matrigel. After one week incubation, the images of the cells were captured using the Olympus CKX41 microscope system.

### Anoikis assay

NCI-N87 and NCI-N87-R cells were cultured in the plates coated with PolyHEMA (Sigma) to avoid the adhesion of cells as described previously [56]. After incubation for 72 h, the cells were harvested and cell apoptosis was measured using Annexin V-fluorescein isothiocyanate (FITC) detection kit (Calbiochem). The experiment was performed for three times.

### Colony-formation assay

Anchorage-independent growth of cells was determined by soft agar colony formation assays. The parental and trastuzumab resistant gastric cancer cells were suspended in DMEM containing 10% FBS and 0.7% agar and plated on top of the bottom layer of 1.2% agar in six-well plates and fed twice a week by adding 0.3 ml medium with or without 5 μg/ml trastuzumab. Colonies were stained with 3-(4,5-dimethylthiazol-2-yl)-2,5 diphenyltetrazolium bromide (MTT), photographed, and measured after 16 days. The experiment was repeated twice.

### Flow cytometric analysis

NCI-N87 and NCI-N87-R cells were stained with the rabbit monoclonal antibody against CD44. After washing with phosphate buffered saline (PBS) for three times, the cells were incubated with FITC-conjugated goat anti-rabbit IgG at 4°C for 30 min. The fluorescence intensity was analyzed by flow cytometry (Becton-Dickinson).

### Cell sphere culture

NCI-N87 or NCI-N87-R cells were collected and washed to remove sera and then suspended in serum-free DMEM/F12 medium containing 100 ng/ml recombinant human epidermal growth factor, 20 ng/ml recombinant human basic fibroblast growth factor, 2% B27 supplement without vitamin A, 1% N2 supplement (Invitrogen), 100 IU/ml penicillin, and 100 μg/ml streptomycin. The cells were subsequently cultured in ultra low attachment six-well plates (Corning) at a density of 1000 cells/well for two weeks.

### Transfection and luciferase assays

To detect the STAT3 activation, NCI-N87 and NCI-N87-R cells were co-transfected with the STAT3 reporter (Promega) and pRL-TK reporter plasmids (Promega) using lipofectamine 2000 (Invitrogen) following the manufacturer's protocol. To test the activities of the Notch signaling, NCI-N87 and NCI-N87-R cells were co-transfected with the Notch luciferase reporter plasmid pGA981-6 (a generous gift from Professor Hua Han, the Fourth Military Medical University of China) and pRL-TK reporter plasmid (Promega). After transfection for 48 h, the cells were lysed in lysis buffer (Promega). Firefly and Renilla luciferase activities were measured with a dual luciferase assay kit (Promega) according to the manufacturer's instructions. All experiments were carried out in triplicate.

### *In vivo* tumor model

Five to six week old male athymic BALB/c nude mice were purchased from Beijing Vital River Laboratory Animal Technology. The mice were divided into two groups randomly and each group contained five mice. A total of 0.1 ml NCI-N87 or NCI-N87-R cell suspension (5 × 10^7^ cells/ml) was injected subcutaneously in the right upper flank of the mice. Forty days following tumor implantation, mice were sacrificed. The livers of the mice were autopsied and fixed in order to evaluate the metastatic potential of NCI-N87 and NCI-N87-R cells *in vivo*. To determine the tumorigenesis of NCI-N87 and NCI-N87-R cells *in vivo*, the mice were divided into three groups randomly and each group contained five mice. 2 × 10^5^, 1 × 10^5^, and 0.5 × 10^5^ NCI-N87 and NCI-N87-R cells were injected subcutaneously in the left or right upper flank of the mice, respectively. Twenty days following tumor cell implantation, the photographs of mice bearing tumors were taken. Alternatively, 2 × 10^5^, 0.5 × 10^5^, or 5 × 10^3^ NCI-N87 and NCI-N87-R cells were injected subcutaneously to the mice.

### Statistical analysis

All data were presented as mean±SD. Student's t test was used for comparison between two groups. For comparison of three or more groups, one-way ANOVA followed by Bonferroni post hoc test was used. *P*<0.05 was considered statistically significant.

## SUPPLEMENTARY MATERIAL FIGURES


