# Supplementary material for: Acquisition of resistance to trastuzumab in gastric cancer cells is associated with activation of IL-6/STAT3/Jagged-1/Notch positive feedback loop
Source: Oncotarget. 2014 Dec 31;6(7):5072–87. doi: 10.18632/oncotarget.3241 (PMC4467134; doi:10.18632/oncotarget.3241)
Supplement: Supplementary file 1 [file oncotarget-06-5072-s001.pdf]

Acquisition of resistance to trastuzumab in gastric cancer cells is associated with activation of IL-6/STAT3/Jagged-1/Notch positive feedback loop

Supplementary Material

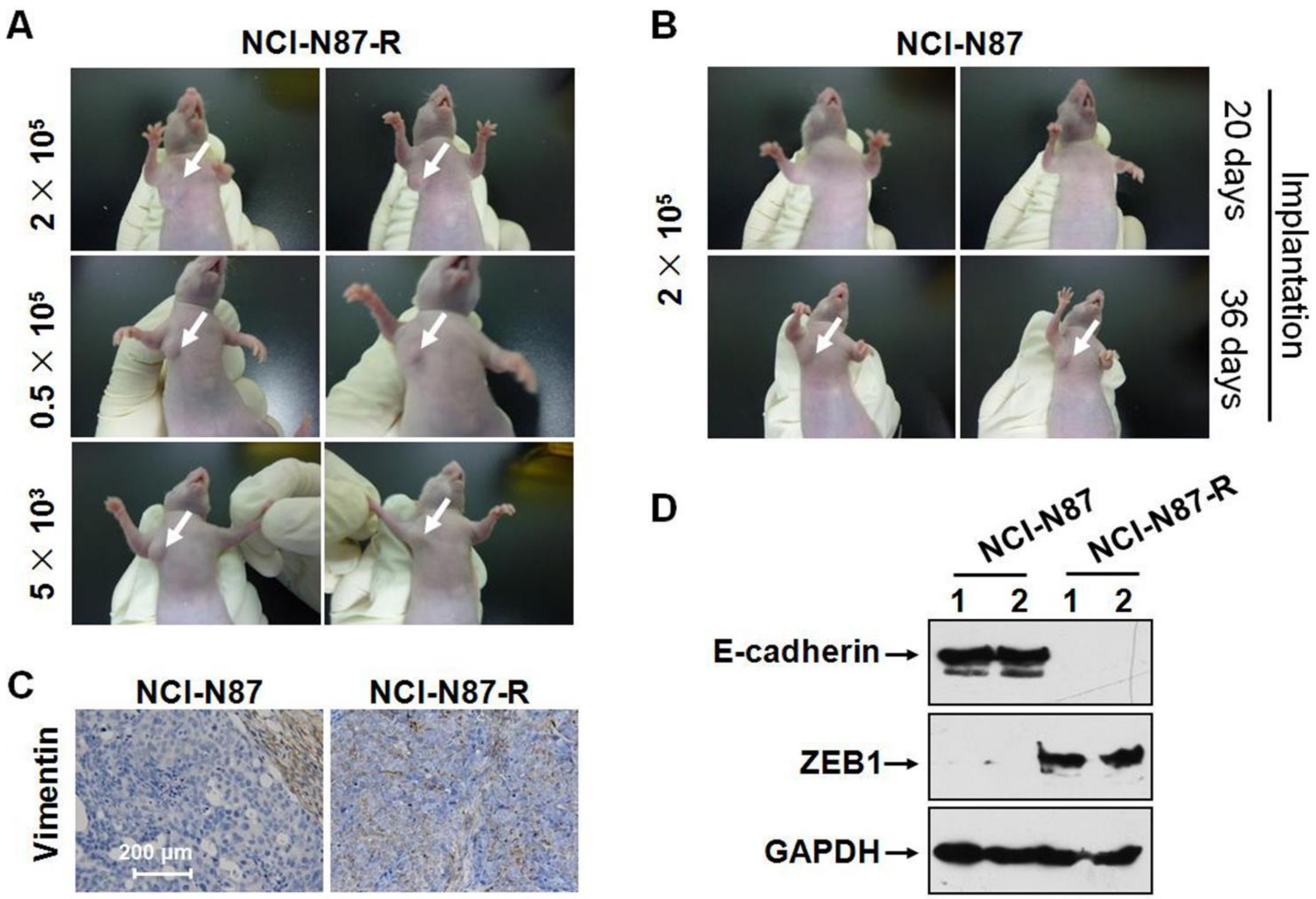

Supplementary Figure S1: A and B, 2×10^5, 0.5×10^5, and 5×10^3 NCI-N87 and NCI-N87-R cells were injected subcutaneously to the mice. 20 (for NCI-N87-R cells) or 36 days (for NCI-N87 cells) following tumor cell implantation, the mice bearing tumors were photographed. C, The expression of vimentin in the xenograft tumor tissues was analyzed by immunohistochemistry. D, The expression of E-cadherin and ZEB-1 in the xenograft tumor tissues was analyzed by Western blot.

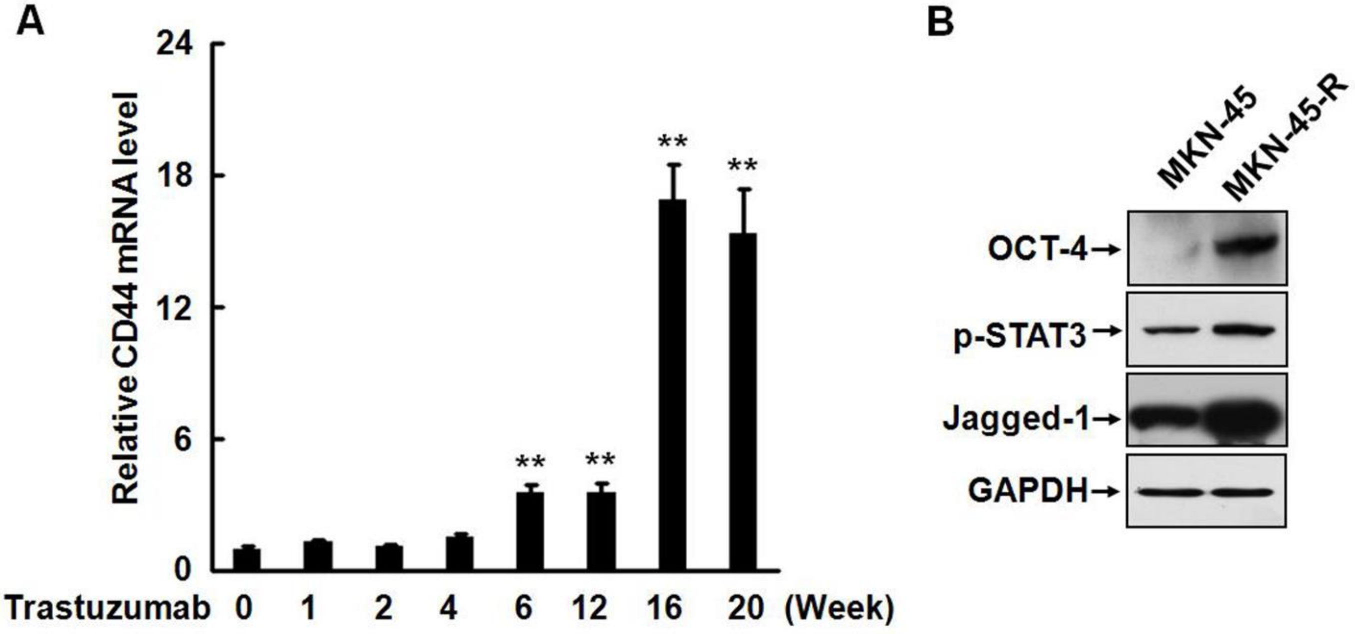

Supplementary Figure S2: A, The expression of the CD44 mRNA was analyzed by real-time RT-PCR at the indicated time points. B, The expression of OCT-4, phosphorylated STAT3, and Jagged-1 was analyzed in MKN-45 and MKN-45-R cells. ** *P*<0.01

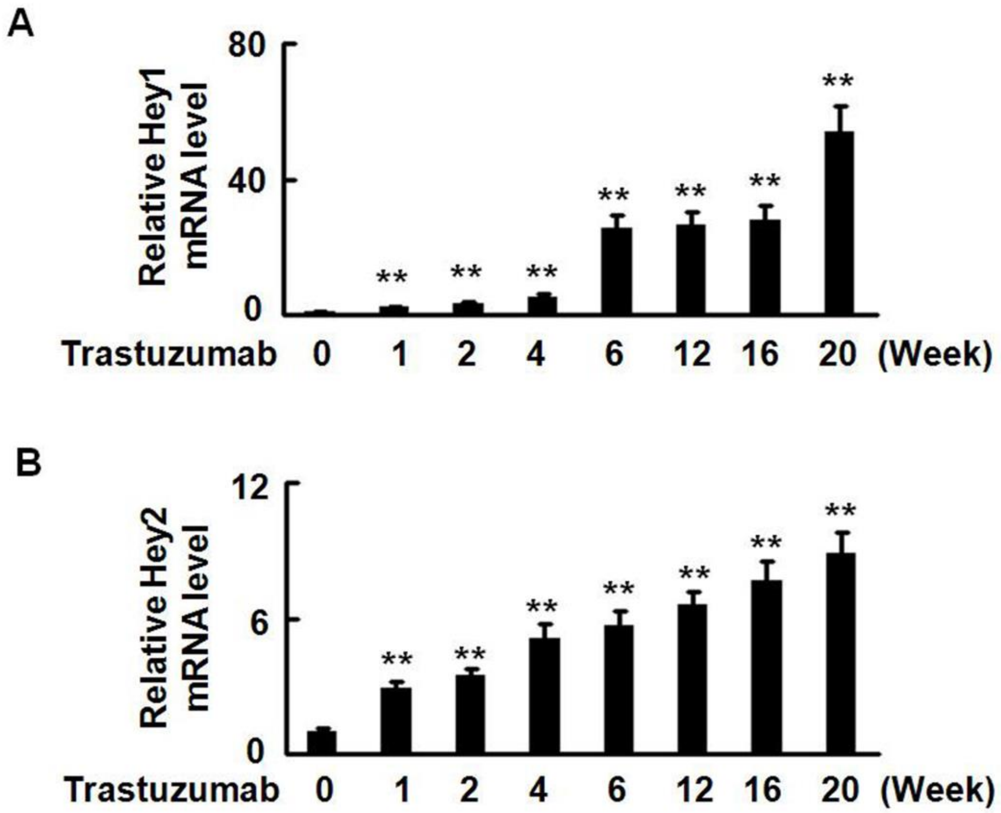

Supplementary Figure 3: A and B, The expression of Hey1 (A) and Hey2 (B) was analyzed by real-time RT-PCR at the indicated time points after trastuzumab treatment.

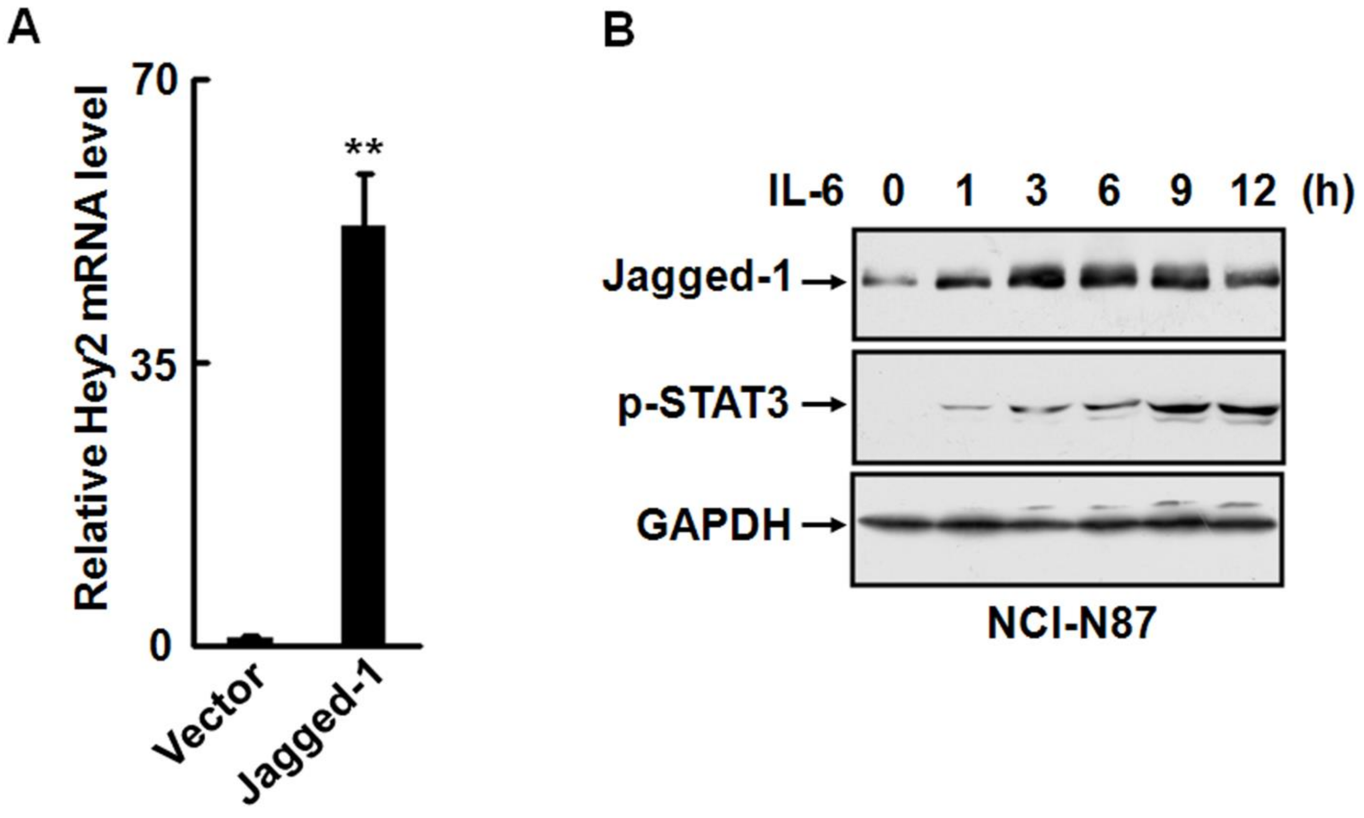

Supplementary Figure 4: A, NCI-N87 cells were transfected with the plasmid expressing Jagged-1. The expression of Hey2 at the mRNA level was analyzed by real-time RT-PCR. B, NCI-N87 cells were treated with 10 ng/ml of IL-6 and the expression of Jagged-1 and phosphorylation of STAT3 was analyzed at the indicated time points. ** $P<0.01$

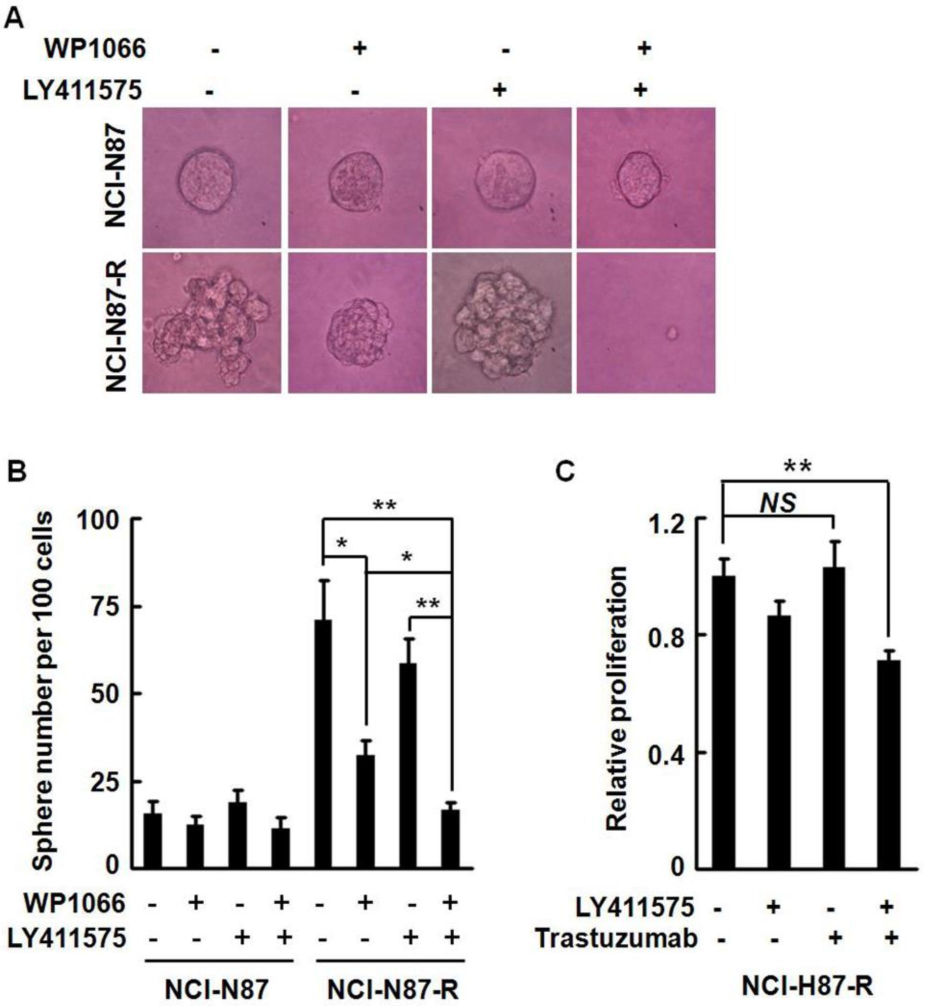

Supplementary Figure 5: A, NCI-N87 and NCI-N87-R cells were suspended in a mixture of Matrigel matrix and culture medium (1:24, v/v) and then layered onto solidified Matrigel in the presence of 2.5 μM WP1066 or 10 μM LY411575 or both. The capacity of the cells to invade through the reconstituted Matrigel matrix was analyzed. B, The *in vitro* self-renewal capacities of NCI-N87 and NCI-N87-R cells were assessed by spheroid colony formation assays by culturing the cells under nonadherent conditions with serum-free media in the presence of 2.5 μM WP1066 or 10 μM LY411575 or both. After two weeks of culture, sphere number per 100 cells was counted. C, The *in vitro* proliferation activities of NCI-N87-R cells were measured by CCK8 assays in the presence of 5 μg/ml trastuzumab and 10 μM LY411575.
